# Supplementary material for: Causal Relationship between Aging and Anorexia Nervosa: A White-Matter-Microstructure-Mediated Mendelian Randomization Analysis
Source: Biomedicines. 2024 Aug 16;12(8):1874. doi: 10.3390/biomedicines12081874 (PMC11351342; doi:10.3390/biomedicines12081874)
Supplement: Supplementary file 1 [file biomedicines-12-01874-s001.zip › Supplementary Figure/Figure S7/Single SNP plot of MR analysis for ubm-b-1926 to AN.pdf]

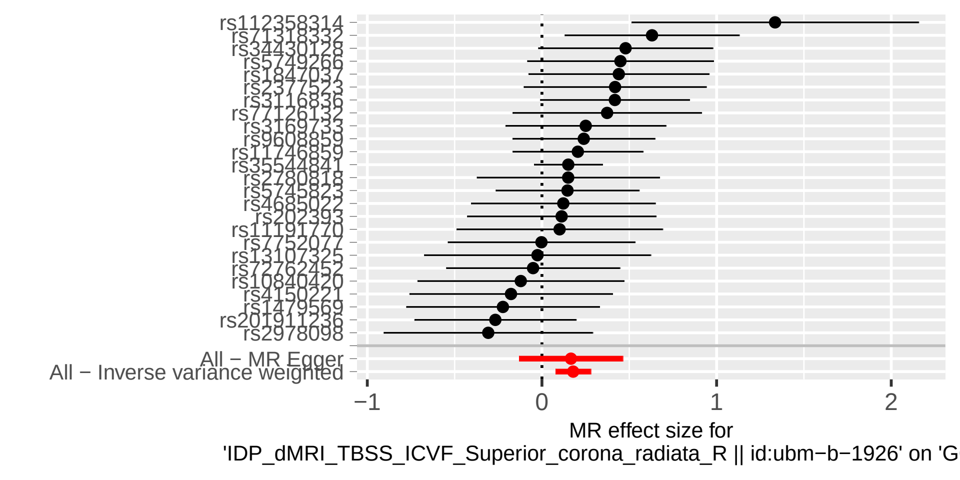
rs112358314
rs71318332
rs34430128
rs5749266
rs1847037
rs2377523
rs3116836
rs77126132
rs3169733
rs9608859
rs11746859
rs35544841
rs2780818
rs5745823
rs4685022
rs202393
rs11191770
rs7752077
rs13107325
rs72762452
rs10840420
rs4150221
rs1479569
rs201911236
rs2978098
All − MR Egger
All − Inverse variance weighted
−1
0
1
2
MR effect size for
'IDP_dMRI_TBSS_ICVF_Superior_corona_radiata_R || id:ubm−b−1926' on 'G
